# Evaluating the Metal Tolerance Capacity of Microbial Communities Isolated from Alberta Oil Sands Process Water

**DOI:** 10.1371/journal.pone.0148682

**Published:** 2016-02-05

**Authors:** Mathew L. Frankel, Marc A. Demeter, Joe A. Lemire, Raymond J. Turner

**Affiliations:** Biofilm Research Group, Department of Biological Sciences, University of Calgary, Calgary, Alberta, Canada; Friedrich Schiller University, GERMANY

## Abstract

Anthropogenic activities have resulted in the intensified use of water resources. For example, open pit bitumen extraction by Canada’s oil sands operations uses an estimated volume of three barrels of water for every barrel of oil produced. The waste tailings–oil sands process water (OSPW)–are stored in holding ponds, and present an environmental concern as they are comprised of residual hydrocarbons and metals. Following the hypothesis that endogenous OSPW microbial communities have an enhanced tolerance to heavy metals, we tested the capacity of planktonic and biofilm populations from OSPW to withstand metal ion challenges, using *Cupriavidus metallidurans*, a known metal-resistant organism, for comparison. The toxicity of the metals toward biofilm and planktonic bacterial populations was determined by measuring the minimum biofilm inhibitory concentrations (MBICs) and planktonic minimum inhibitory concentrations (MICs) using the MBEC ™ assay. We observed that the OSPW community and *C*. *metallidurans* had similar tolerances to 22 different metals. While thiophillic elements (Te, Ag, Cd, Ni) were found to be most toxic, the OSPW consortia demonstrated higher tolerance to metals reported in tailings ponds (Al, Fe, Mo, Pb). Metal toxicity correlated with a number of physicochemical characteristics of the metals. Parameters reflecting metal-ligand affinities showed fewer and weaker correlations for the community compared to *C*. *metallidurans*, suggesting that the OSPW consortia may have developed tolerance mechanisms toward metals present in their environment.

## Introduction

Industrial wastewater has become a pervasive issue in the modern world. Anthropogenic activities, such as mining, introduce and concentrate organic and inorganic contaminants from source or bodies to the surrounding environment [1]. We wanted to explore the hypothesis that microbes from industrial wastewaters would have metal tolerances reflective of the environment from which they were sourced. To this end, we evaluated an endogenous microbial community inoculated from the wastewater of a Canadian oil sands extraction operation for our proof of principle.

The accumulation of waste tailings from surface mining has made water treatment [1,2] and management [3,4] and increasing issue for the Canadian oil sands industry. The process of extracting bitumen is water intensive [3,4], resulting in large volumes of tailings waste consisting of clay, sand, polycyclic aromatic hydrocarbons, residual bitumen (heavy oil), naphthenic acids, diluents, and heavy metals [1,2,5]. This mixture separates into particulate mature fine tailings (MFT) topped with oil sands process water (OSPW) [1,2]. Environmental concerns regarding OSPW have resulted in their storage in end pit lakes, as they are subject to a “zero discharge” policy [2]. Reclamation is the industry standard for tailings management, relying heavily on remediation by the natural ecology to detoxify OSPW of organic contaminants [1,2]. The timeframe for this remediation is variable and uncertain [1,2], and can be further complicated by the presence of metals [6]. Metals have been known to inhibit a range of microbial processes [7], including those involved in the degradation of organic pollutants [6]. Some metals reported to be present in OSPW include: Al, As, Cd, Cr, Cu, Fe, Ni, Zn–many at concentrations well above ranges deemed safe by environmental regulatory agencies [1,2]; see S1 Table for metals concentrations reported [8–10] in tailings ponds and process affected waters. To reduce wastes and potential environmental impacts, Canada’s oil sands operations recycle wastewater [1,3,4,11]. While reducing the demand for fresh water, this recycling results in the concentrating of trace metals in tailings ponds [1].

Many metals are essential to biological systems, as they participate in biochemistries not attainable by organic molecules alone; others are nonessential and can be toxic at low concentrations [12]. In excess, metals can disrupt natural biogeochemical processes [13], and at high enough concentrations all metals can impose toxicity [14]. As a result, many microbes have evolved mechanisms to survive and thrive in metal-rich environments; as reviewed by Harrison *et al*. [15] and Lemire *et al*. [16]. Recently, biofilms have become the focus of research related to metal–microbe interactions due to their increased capacity to tolerate many metals [15,17–20].

Environmental bacteria are commonly found associated in biofilms, where a microbial community is attached to a surface and embedded in a self-secreted layer of extracellular polymeric substances [21]. Biofilms can be single- or multispecies and the close proximity of cells within the matrix provides opportunity for exchanges such as gene transfer via plasmids [22], co-metabolism, and synergism [19,20]. As a result, cellular specialization in biofilms [15] can result in metabolic and physiological adaptations and responses not seen in their planktonic counterparts [23], in turn providing protection from environmental extremes [21]. Thus, microbial biofilms have become the target of research and technologies for applications such as wastewater treatment [24], microbial influenced corrosion [25], and biomining [26].

Our research group has observed that *in situ* multispecies communities from OSPW [27] and MFT [28] can be cultured *in vitro* directly as a biofilm capable of biomineralizing metals [29] as well as degrading commercially available naphthenic acids [30]. The purpose of this study was to investigate the tolerances of a microbial community cultured from OSPW to different metal stresses. We compared the response of the OSPW consortia we have cultured [27] to that of *Cupriavidus metallidurans* (CH34), a model metal-resistant organism known to have a variety of overlapping metal efflux systems [12,31,32]. The MBEC ™ assay was used to evaluate the OSPW community and *C*. *metallidurans’* minimum inhibitory concentrations of 22 metal ions to biofilm (MBIC) and planktonic (MIC) cultures. In addition, we also correlated our tolerance data with physicochemical characteristics of each metal tested to provide insight into tolerance and toxicity in the OSPW community. In the present study, the planktonic inoculant was introduced directly to metal challenged media. Rather than of challenging pre-established biofilms as commonly performed in previous studies [17,33–36], our approach allows for the assessment of cell attachment and biofilm growth under metal stress, which is more reflective of environmental conditions.

## Methods and Materials

### Bacterial inoculants and media

In all experiments, the microbial community was sourced from an open pit OSPW sample from an oil sands tailings pond in northern Alberta, Canada [27]. Acquisition of the OSPW sample was performed by an oil sands operator and shipped to the University of Calgary. Though the chemical and biological composition of the OSPW sample likely changed throughout the handling and storage process, OSPW samples were nonetheless stored in a sealed vessel at 4°C [37] in order to minimize these effects. Our comparator organism, *C*. *metallidurans* strain CH34 (ATCC^®^ 43123), was purchased from Cedarlane Laboratories. Culturing procedures are outlined in the metal susceptibility testing section.

Metal susceptibility assays were conducted using an adapted Bushnell-Haas (BH) minimal salts media [38] (pH 6.6, 1.0g KH_2_PO_4_, 1.0g Na_2_HPO_4_, 0.5g NH_4_NO_3_, 0.5g (NH_4_)_2_SO_4_, 0.2g MgSO_4_•7H_2_O, 0.02g CaCl_2_•2H_2_O, 0.002g FeCl_3_, 0.002g MnSo_4_•2H_2_O, 1g of yeast extract (ICN Biomedica’s, Inc., catalogue number 103303, lot number 90621), per litre of double distilled water). BH has been used to evaluate hydrocarbon utilization and biodegradation by microbes [14,38] as well as in previous work to evaluate OSPW communities [27,39,40]. We maintained a slightly acidic (pH 6.6) media to retain metals as bioavailable free ions, as compared to hydroxyl-metal complexes formed under alkaline (>7.5) conditions [1,6]. BH is a phosphate-buffered media; as phosphate is known to react with metals [41], a number of chemical interactions could have occurred, potentially decreasing the bioavailability of metal ions and/or essential nutrients. These media affects can lead to considerable differences in metal tolerances observed in our study versus others. Irrespective of these concerns, our *C*. *metallidurans* data supports previous research of metal toxicity in this organism [31] (see S2 Table), which allowed us to use it as a reliable comparator under the conditions used here.

Controls for inoculum viability were performed in tryptic soy broth. Subcultures, planktonic minimum inhibitory concentrations (MICs), and minimum biofilm inhibitory concentrations (MBICs) were performed on tryptic soy agar (TSA) plates. TSA plates for subcultures, MICs and MBICs, as well as metal susceptibility cultures were incubated at 25°C (following previous OSPW microbial work [27–30]), as this was a proof of principle to establish the metal tolerances, rather than an *in situ* study, of the indigenous OSPW microbiota. To minimize the effects of metal carryover to agar plates [36], a universal neutralizer was employed after the metal challenge in recovery steps (see Metal susceptibility testing).

### Stock metal solutions

Metal ions were provided in various salt forms, which can be found on S3 Table. While some metals ionize to their cationic species, other redox active metals (V, Mo, W, Te, As) form oxyanions in solution [42]. Prioritization of pollutants by the Canadian Environmental Protection Act [43] and the U.S. Environmental Protection Agency [44] directed the choice of metals used in the study. Other more toxic metals (e.g. Ag, Te) were also included to gain a wider perspective of metal tolerances and susceptibility.

Stock metal solutions were made at 1M (or the highest soluble concentration) in ddH_2_O, filter sterilized (0.45μm) and stored in sterile glass vials, at room temperature. Immediately prior to each assay, the stock was diluted 1:1 with media.

### Metal susceptibility testing

Metal susceptibility testing of the OSPW mixed species and *C*. *metallidurans* were assayed using the Calgary Biofilm Device (CBD) with a protocol modified from Harrison *et al*. [33]. In this process, a CBD peg lid is placed in a standard 96-well microtiter plate, on which biofilms adhere to polystyrene pegs. Briefly, the challenge plate was prepared by serially-diluting the stock metals two fold along the rows of the microtiter plate with BH media, to a final volume of 75μL. Each well of the plate was then directly inoculated with 75μL of OSPW or a 1:15 dilution of a 1.0 McFarland Standard (approx. 3 x 10^8^ cfu mL^-1^) suspension of *C*. *metallidurans*. With this method, planktonic attachment and biofilm growth were established under metal challenge, rather than exposing a mature biofilm to antimicrobials as with previous susceptibility work [17,33–36]. Following 2 days for the OSPW consortia, and 1 day for *C*. *metallidurans*, biofilms cultures were replenished with 150μL of fresh challenge media. While this provided growing biofilms with fresh resources, this also rids the system of planktonic cells, ensuring toxicological effects and/or survivorship to metal challenges were only related to the presence of the biofilm on the peg [27]. With the environmental biofilms, this also eliminated any metals or other conflicting contaminants within the OSPW inoculum. Once replenished, environmental biofilm cultures were incubated for an additional 4 days (2 days for *C*. *metallidurans*) at 25°C, and 125 rpm; our unpublished OSPW mixed species growth assays (assessed via measuring protein content) suggests that 6 days are required for a robust environmental biofilm to form under similar conditions. Since planktonic media cannot simply be replaced without losing all cells in the process, 20μL of spent biofilm media served as the planktonic inoculum. Upon introduction to fresh challenge plates with 130μL of growth media containing the appropriate corresponding metal challenge, planktonic cultures were incubated at 25°C and 125rpm, for a total of 6 and 3 days for OSPW and *C*. *metallidurans*, respectively. After exposure to metal challenges, biofilms were rinsed twice with 0.9% saline and sonicated for 10min (VWR International, Aquasonic model 250HT) off pegs into a ‘recovery plate.’ Recovery plates contain 0.9% saline with 0.1% Tween-20 and a ‘universal’ neutralizer (UN) [1.6 mM L-histidine, 1.6 mM L-cysteine, and 2.1 mM reduced glutathione] [36]. A similar plate (sans Tween-20) was also made for planktonic recovery to which aliquots are added from planktonic challenge plates, yielding a 1:10 dilution. Aliquots (20 μL) from recovery plates were then spotted on TSA plates, incubated, and scored. Inhibition of growth was assessed as +/-, where ≥ 50% spot area growth constituted uninhibited growth (+) and < 50% reflected inhibition (-) at the given metal concentration. If no growth was evident, the stock metal solution was diluted and tested over a lower range; in the present study these treatments ranged from 2.5x10^-7^–2.5x10^2^ mM.

### Metal ion parameters and statistical analysis

Physicochemical parameters used in this study can be found on S3 Table. Electronegativity (X_m_), standard reduction-oxidation potentials (ΔE_0_), and first ionization energy (*I*_*1*_), Pearson’s softness index (σ_p_), metal-sulfide solubility product (pK_sp_), and the log of the first hydrolysis constants (|Log K_OH_|) were correlated with the logarithm of MIC and MBIC values using linear regression plots. Linear regression plots and coefficients of determination (r^2^) were obtained using GraphPad Prism version 6.0. P-value of less than 0.05 was used to determine line slopes that were significantly non-zero. Heatmaps and hierarchical analysis were made with RStudio version 0.98.977 using Heatmaps.2 from the ‘gplots’ package.

## Results

### OSPW consortia and *C*. *metallidurans* metal tolerances

**Fig 1:**
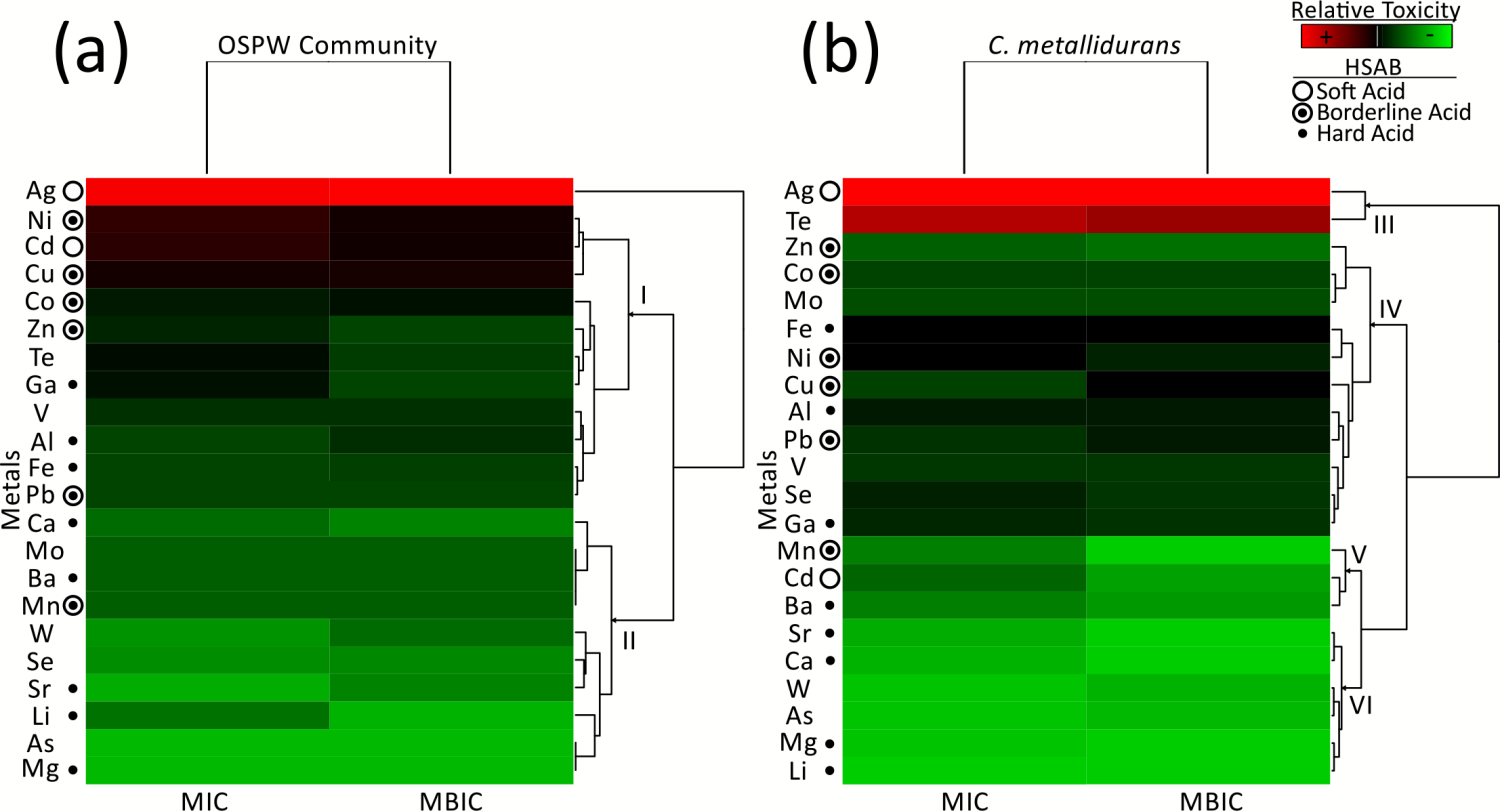
Heat map analysis of relative metal toxicity to (a) the OSPW consortia and (B) *C*. *metallidurans*. The heat map colors represent average minimum inhibitory concentrations (MIC) and minimum biofilm inhibitory concentrations (MBIC) based on average values obtained from two to nine trials, where red reflects the most toxic metals and green represents the least toxic. The Hard Soft Acid Base (HSAB) designation describes the behaviour of metal ions based on preferential donor ligands. Soft acids prefer to bind with thiol (S-group) ligands, hard acids with N and O, and borderline acids have varied preference for S, N, and O-containing ligands.

Tolerance data is displayed as a heat map (with hierarchical clustering) in Fig 1 for ease of evaluation and comparison between conditions, metal inhibitory values can be found in S2 Table. Of the 22 metal ions tested, As and Mg were not inhibitory to the OSPW community’s growth at the highest concentrations used in the assay (250 mM), regardless of mode of growth (planktonic vs. biofilm). This contrasts that of *C*. *metallidurans* growth, whose planktonic inhibitory concentrations were not reached for Li, Ca, or Mg. Ag proved to be most toxic metal to both the OSPW consortia and *C*. *metallidurans*, with inhibitory concentrations of ≤1.0 μM for planktonic and biofilm cultures. Overall the OSPW community exhibited comparable metal tolerance levels to *C*. *metallidurans*, regardless if grown as planktonic or biofilm cultures.

Hierarchical clustering of metal tolerance data demonstrated nonessential metals (Ag, Te, Cd) to be among the most toxic and dominated by thiophillic HSAB soft acids [45]. These generally grouped most distant from the rest: Ag for the OSPW community (Fig 1A); Ag and Te for *C*. *metallidurans* (Fig 1B, node III). Tellurite (TeO_3_^2-^), while not considered a soft acid, has been suggested to oxidize cellular sulphur [46], further underscoring the role of sulphur affinity in metal susceptibility. Borderline acids, which can be thiophillic and include micronutrients (Ni, Cu, Co, Zn, Fe, Mn,) [13,45], were found to be amongst the most toxic (top 27%, excluding Pb and Mn) to the OSPW community. Most borderline acids clustered together, with hard acids being more distantly associated (Fig 1A, node I). This was less pronounced with our model organism (Fig 1B, nodes IV and V), but not unexpected due to *C*. *metallidurans’* reported resistance to thiophillic metals (specifically Cd, Zn, Ni, and Co) [12,31]. Hard acids, which include essential metals (Ca, Mg), clustered with the least toxic metals (Fig 1A, node II; Fig 1B, node VI).

### Correlations with physiochemical characteristics

In order to assess whether the OSPW consortia and *C*. *metallidurans* demonstrated a similar sensitivity to metal challenges, we investigated how the inhibitory concentrations of the metals correlated with the metals’ physicochemical characteristics. Here we plotted the ion-specific physicochemical parameters of our assayed metals (S3 Table) against the logarithm of our MIC and MBIC values (S2 Table), excluding Mg since it was not found to be toxic at the highest concentrations assayed. The parameters investigated in this study included: electronegativity (X_m_), first ionization energy (E_I_) (previously known as ionization potential, ΔIP), the negative logarithm of the sulfide-metal solubility constant (pK_sp_), and the absolute value of the log of the first hydrolysis constant of the metal hydroxide (|Log K_OH_|). These physiochemical characteristics (S3 Table) reflect physical and chemical properties of the metals ions, which explain their ionic behaviour in solution as well as their reactive and complexation properties, providing clues to their mechanisms of toxicity in bacteria as well as metal-specific responses. We chose these in particular, as others have described similar linear relationships between the chosen parameters and metal toxicity in other taxonomies [47–51] as well as with single species microbial work [33]; we wanted to evaluate how this translated to an environmental microbial community as a whole.

**Fig 2:**
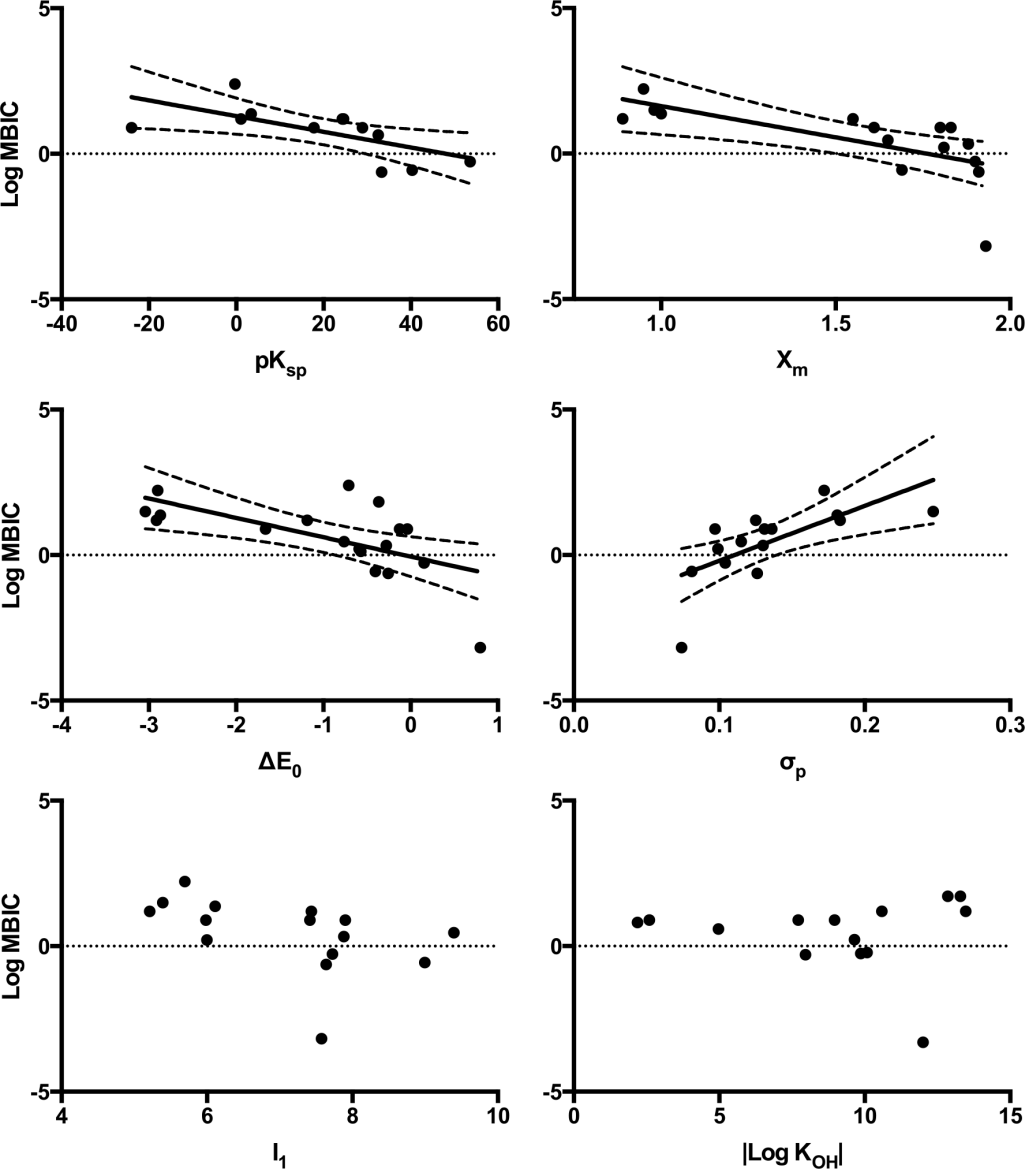
Linear regression analysis of MBIC values plotted against physicochemical parameters for OSPW consortia. MBIC correlations with physicochemical parameters illustrate a typical metal susceptibility profile of the OSPW community. Parameters include: metal-sulfide solubility product (pK_sp_), electronegativity (X_m_), standard reduction-oxidation potentials (ΔE_0_), Pearson’s softness index (σ_p_), first ionization energy (*I*_*1*_), and first hydrolysis constant (|Log K_OH_|). Trend lines and 95% confidence bands (dashed lines) shown on linear regressions that correlate with significance.

**Fig 3:**
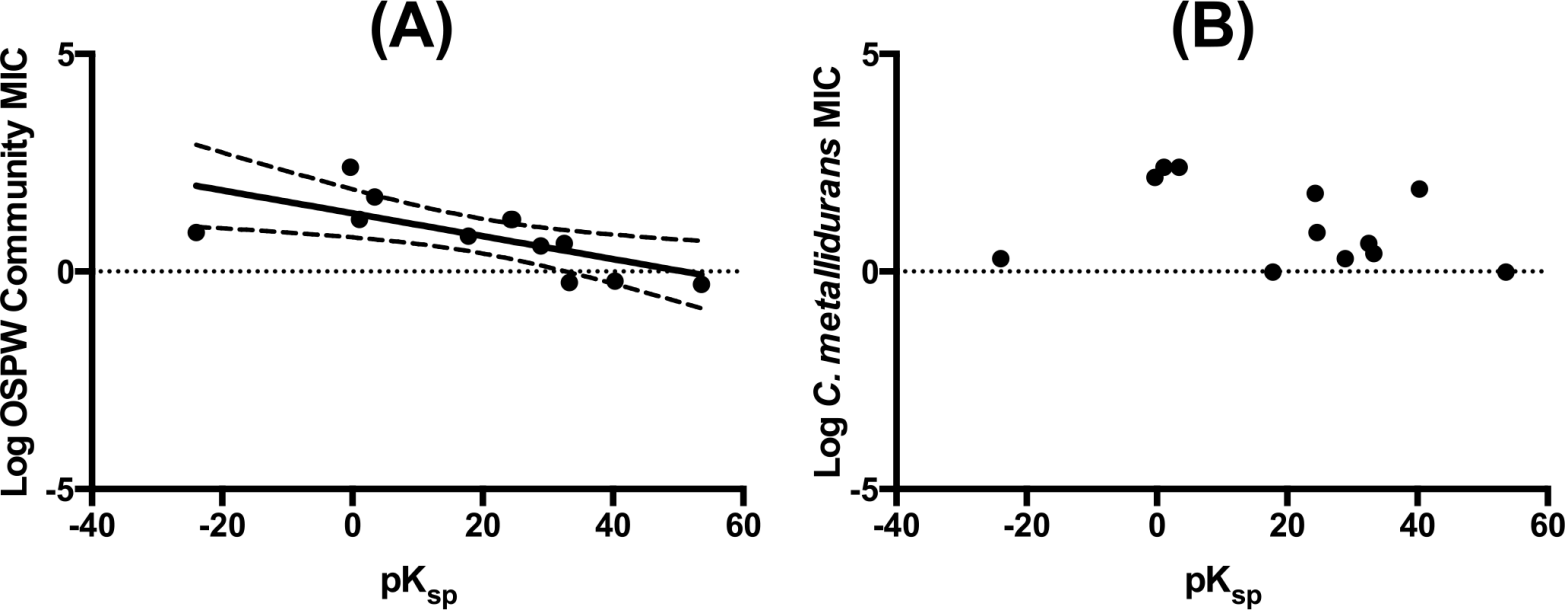
Planktonic minimal inhibitory concentrations plotted against pK_sp_. Significant correlation is evident with the OSPW community (A), and absent with *C*. *metallidurans* (B). Dashed lines show 95% confidence bands.

Linear regression was used to analyse physicochemical parameters against the logarithm of MIC and MBIC values. A typical correlational profile of the OSPW community versus each of the parameters evaluated can be found in Fig 2. ΔE_0_, X_m_, and σ_p_ showed statistically significant correlations for both the OSPW mixed species and *C*. *metallidurans* across form (planktonic vs. biofilm) (Table 1); I_1_ and |Log K_OH_| demonstrated no correlations. pK_sp_ was the differentiating parameter between the organisms tested, where significant correlations were evident with the OSPW community, while absent with *C*. *metallidurans* (Fig 3). Analysis of these correlations drew our attention to the observation that stronger correlations with better fits were obtained with inhibitory values of our single species model organism, *C*. *metallidurans* (Table 1).

**Table 1:** Linear regression analysis of physicochemical parameters with metal toxicity values for OSPW cultures and *C*. *metallidurans*, represented as minimum inhibitory concentrations (MIC) and minimum biofilm inhibitory concentrations (MBIC).

	OSPW Community				*C*. *metallidurans*			
	MBIC		MIC		MBIC		MIC	
Parameter	r^2^	P-value	r^2^	P-value	r^2^	P-value	r^2^	P-value
pK_sp_	**0.43**	0.021	**0.47**	0.013	0.12	0.275	0.10	0.317
ΔE_0_	**0.38**	0.007	**0.40**	0.005	**0.42**	0.004	**0.47**	0.002
I_1_	0.22	0.075	0.20	0.091	0.10	0.242	0.08	0.317
Χ_m_	**0.44**	0.007	**0.43**	0.008	**0.47**	0.005	**0.49**	0.004
σ_p_	**0.46**	0.006	**0.51**	0.003	**0.47**	0.005	**0.39**	0.012
|Log K_OH_|	0.00	0.981	0.00	0.838	0.05	0.461	0.09	0.311

Values in bold denote a significant correlation (P<0.05).

### Correlations of metal toxicity based on electron shell classifications

Metal ions have been described and classified by a variety of systems. In this study we adopted the scheme described by Kaiser [52], which classified metal ions based on outer shell structure (S3 Table). Class I includes ions with completely filled *p* orbitals, this included those found in the first three groups of the periodic table. Class II ions have partly or completely filled *d* orbitals, which include the transition metals and are classically known as the *heavy metals*. Class III ions have completely filled *s* orbitals, including elements from group 13–16 on the periodic table. When compared to Fig 1, it is apparent that on average Class I (Li, Mg, Ca, Sr, Ba, Al, Ga) and Class III (Pb) ions were less toxic than Class II ions (Fe, Ag, Cd, Mn, Co, Ni, Cu, Zn). It was found that when only Class I ions were used for linear regressions, few correlations were seen with the OSPW community (σ_p_ (MIC), ΔE_0_ (MBIC) and X_m_ (MBIC)), while more were obtained for *C*. *metallidurans* across form with ΔE_0_, X_m_, and |Log K_OH_| (and σ_p_ with MBIC) (Table 2). When only Class II ions were used for linear regressions, fewer significant correlations were found, most being ΔE_0_ for both the OSPW consortia and *C*. *metallidurans* (Table 3). Only one of the metals from this study was from class III, thus no linear regression could be made.

**Table 2:** Linear regression analysis of physiochemical parameters with the OSPW community and *C*. *metallidurans* metal toxicity values using class I metal ions, represented as minimum inhibitory concentrations (MIC) and minimum biofilm inhibitory concentrations (MBIC).

	OSPW Community				*C*. *metallidurans*			
	MBIC		MIC		MBIC		MIC	
Parameter	r^2^	P-value	r^2^	P-value	r^2^	P-value	r^2^	P-value
pK_sp_	0.77	0.321	0.85	0.251	0.73	0.346	0.69	0.379
ΔE_0_	**0.72**	0.034	0.54	0.096	**0.76**	0.023	**0.76**	0.023
I_1_	0.11	0.512	0.14	0.460	0.17	0.412	0.16	0.436
Χ_m_	**0.67**	0.047	0.54	0.096	**0.79**	0.018	**0.83**	0.011
σ_p_	0.42	0.167	**0.74**	0.028	**0.74**	0.027	0.63	0.061
|Log K_OH_|	0.68	0.086	0.67	0.090	**0.86**	0.024	**0.86**	0.024

Values in bold denote a significant correlation (P<0.05).

**Table 3:** Linear regression analysis of physiochemical parameters with the OSPW community and C. metallidurans metal toxicity values using class II metal ions, represented as minimum inhibitory concentrations (MIC) and minimum biofilm inhibitory concentrations (MBIC).

	OSPW Community				*C*. *metallidurans*			
	MBIC		MIC		MBIC		MIC	
Parameter	r^2^	P-value	r^2^	P-value	r^2^	P-value	r^2^	P-value
pK_sp_	0.72	0.068	**0.87**	0.020	0.03	0.767	0.24	0.404
ΔE_0_	**0.63**	0.018	**0.71**	0.008	**0.71**	0.009	**0.83**	0.002
I_1_	0.02	0.716	0.07	0.527	0.14	0.364	0.11	0.426
Χ_m_	0.32	0.142	0.37	0.109	0.40	0.094	**0.52**	0.045
σ_p_	0.42	0.082	0.42	0.046	0.32	0.141	0.31	0.155
|Log K_OH_|	0.26	0.192	0.24	0.195	0.03	0.665	0.01	0.843

Values in bold denote a significant correlation (P<0.05).

## Discussion

### OSPW consortia and *C*. *metallidurans* metal tolerances

A bacterial community obtained from a particular environment is typically considered to have evolved for growth in the conditions of said environment [53]. This principle likely applies to our metal resistant model organism, *C*. *metallidurans* (CH34), which was isolated from a metal processing factory [12] and has been reported to have resistance to a variety of metals [12,31]. The question in our study evaluates if a community of microbes from a rapidly produced new environment (i.e. mining operations) would have tolerances reflective of the increasingly harsh conditions. In the case of OSPW, an environment reported to have a wide diversity of organic and inorganic contaminants [1,2], we observed this community to exhibit comparable tolerances to metal challenges as *C*. *metallidurans*; tolerance data is displayed as a heat map in Fig 1 for ease of evaluation, metal inhibitory values can be found in S2 Table. The inhibitory concentrations of the OSPW multispecies communities were higher than single species work done by others using similar parameters [17,33,34]. Both the OSPW community and *C*. *metallidurans* displayed tolerance to metal concentrations above what has been reported in OSPW [8–10]. The OSPW consortia demonstrated greater metal tolerances than *C*. *metallidurans* to some of the more abundant metals found in tailings ponds and process affected waters (Al, Fe), as well as others present at lower concentrations (Mo, Pb) (S1 Table).

### Metal physicochemical characteristics and toxicity

The correlations demonstrated in this study suggest that microbial metal toxicity is determined, to some degree, by the inherent physicochemical characteristics of the metal ions [33]. What these physicochemical parameters (X_m_, ΔE_0_, *I*_*1*_, σ_p_, pK_sp_,|Log K_OH_|) have in common is their reflection of a metal’s affinity for electrons and/or ligands. Preferences to biologically significant ligands can effect protein folding and function, change reduction potential of essential chelating metals, and facilitate the co-transport of metals into cells [16]. Others have demonstrated similar correlations between X_m_ and metal toxicity across taxonomical kingdoms [33,47,48]. Some suggest that metal ions exert their toxicity by tight bonds to cell surface exposed biochemical ions [54], which is related to electronegativity differences between ions.

ΔE_0_, also known as reduction-oxidation (redox) potential is a measure of an ion’s ability to donate or accept electrons [52]. The importance of redox-reactive metal species is highlighted by studies demonstrating that some essential metals, including Fe, Cr, Cu, V can undergo redox cycling to fulfil required cellular chemistries [55]. These metals have also been demonstrated to participate in reactive oxygen species (ROS) production when their homeostasis is disrupted [55]. Microbial accumulation of ROS has been linked to the inhibition of vital metabolic enzymes and DNA damage–hindering cell growth and triggering cell death [56].

The softness index (σ_p_) is based on Pearson’s [57] hard and soft acid base (HSAB) theory. HSAB theory classifies metal ions based on preferential biological donor ligands: hard acids prefer to bind with hard bases such as oxygen and nitrogen, while soft acids prefer soft bases, mostly sulfur; borderline ions showing varied preference between the three [49]. With regards to σ_p_, larger values classify ions as ‘hard’, and smaller values are considered ‘soft.’ We found that smaller (soft) values correlated with increased toxicity, in both our environmental and model organisms, as planktonic and biofilm forms. This type of correlation suggests the important role of soft ligands, like reduced sulfur (particularly glutathione), in mitigating metal toxicity and also as part of the antioxidant defense network of the cell [58]. While this type of correlation has been observed in single species metal toxicity work [33,49,50], our results suggest that environmental communities as a whole react similarly to soft metals, which is not expected as past literature suggests mixed species communities (especially as biofilms) would be expected to display different properties and be more resistant to metal toxicity (see Single versus multispecies).

The sulfide-metal solubility product constant (pK_sp_) reflects a metal’s tendency for S bonding [59]. This parameter has been linked with enzyme inhibition [41] and even toxicity in aquatic organisms [47]. pK_sp_ correlated very well with the OSPW community (Table 1). While this affinity for sulphur is important for toxicity in the OSPW consortia, there are no correlations with our model organism. *C*. *metallidurans* has plasmids specifically associated with thiophillic metal resistance [12,31]. This predisposed soft-metal resistance and known metal efflux systems in *C*. *metallidurans* [12,31] resolves the disparity in pK_sp_ correlations between and our model and OSPW cultures, suggesting that metal ion efflux is less prevalent in OSPW microbes.

The log of the first hydrolysis constant, |Log K_OH_| (K_OH_ for M^n+^ + H_2_O → MOH^n-1^ +H^+^), can be used as a proxy to show metal ion affinity towards hard bases, such as those with oxygen donor atoms [50]. These include organic functional groups that are vital to protein structure and function and can be found throughout an organism [51]. Lower |Log K_OH_| correlated with higher toxicity, and demonstrated strongest correlations with *C*. *metallidurans* under Class I metal stress. Considering |Log K_OH_| reflects an affinity towards hard bases, it is not surprising that Class I metals, which comprise of entirely hard acids (S3 Table), would correlate with this parameter. The lack of correlation with the OSPW community may suggest a defence mechanism to toxicity by Class I metals.

Our findings shared some similarities and differences to findings by Workentine *et al*. [33], which evaluated metal ion stress to the soil bacterium *Pseudomonas fluorescens*. As with this study, class II metal ions were found to be more toxic than class I and III metals. On the other hand, Workentine *et al*. found pK_sp_ and ΔE_0_ to correlate strongly with planktonic values but not biofilm. Our study found this was only the case with pK_sp_ for the OSPW consortia class II metals, and the parameter did not correlate at all with *C*. *metallidurans* (Table 1). Also, while the *P*. *fluorescens* study did not find any correlations with class I ions, we observed these metals also contributed to correlations in both our OSPW and *C*. *metallidurans* cultures. Our findings did corroborate the assertion that ΔE_0_ was the only consistent correlating parameter with class II ions, both for the single and multispecies planktonic and biofilm growth modes. These observations were suggested as an indicator to which properties contribute to the overall toxicity of metals [33].

### Growth modes: biofilm versus planktonic

Correlations seen between metal-ion-specific physicochemical parameters (Tables 1–3) were different for biofilm and planktonic cells. This was evident in both the strength of relationships (e.g. σ_p_, ΔE_0_, pK_sp_ (OSPW community), Table 1), and as one growth form having statistically significant correlations that were absent in the other (e.g. σ_p_, ΔE_0_, X_m_ (OSPW community), Table 2). Contrasts in correlations suggests differential mechanisms of tolerance and toxicity between planktonic and biofilm growth forms. This supports earlier observations made by Harrison *et al*. [60], and is grounded in the argument that biofilm physiology is different than planktonic [23,33].

In biofilm cultures, planktonic cells from the OSPW inoculant would have to tolerate metal challenges long enough to initiate attachment [18]. The environmental stress that triggers this attachment response also leads to physiological changes and biofilm formation [61]. Booth *et al*. [23] demonstrated that *P*. *fluorescens* displays distinctly different metabolic responses to Cu stress, whether it was exposed as biofilm or planktonic populations. This highlights an experimental difference that relates to exposure of environmental stress: how an organism tolerates chronic toxin exposure and still proliferates versus receiving a sudden pulse of toxins. Both scenarios exist when we consider pollution release. Here we grew our biofilms under metal duress, as opposed to challenging an established biofilm grown without stress. The method of metal challenging was chosen to assess the ability of the OSPW community to tolerate and grow under continuous metal stress. This study is one of the few to use this approach for microbe metal susceptibility and allows a different perspective than the acute exposure studies investigated by others [17,33–36].

### Single versus multispecies

Considering the prevalence of metals in the environment from which our inoculum was sourced, we expected the multispecies cultures to be as tolerant to metals or better than the single species. As discussed, the OSPW community demonstrated comparable tolerances to our single species model organism, the known metal-resistant *C*. *metallidurans*. However, linear regressions plotted between metal tolerance data and physicochemical characteristics of the metals revealed differences when the correlations of single and multispecies were compared. Stronger correlations were observed with the single species organism compared to the OSPW consortia, as measured by higher r^2^ values of significant relationships (Tables 1–3), may suggest that the endogenous microbial community has developed different adaptations to mediate the toxic effects of metals present in their sourced environment compared to the single species (mostly efflux transporters). Most multispecies biofilm work done to date relates to marine sediment, medical, and industrial bioreactors (as reviewed by Yang *et al*. [62]), yet little work has been done comparing single and multispecies bacterial cultures and their metal tolerances, let alone from an environmental inoculum that would help us rationalize our observations. Barrangue *et al*. [63] exposed environmental mono and multispecies algal biofilms to Cu. Physical composition of biofilms was concluded to bear more weight on Cu sensitivity than species composition, where thicker biofilms conferred higher tolerances. The translatability should be taken with care, as these biofilms were mostly composed of diatoms. Though, this principle has been supported with bacterial biofilm work by many who argue that the biofilm architecture may restrict movement of oxygen, nutrients, and xenobiotics (e.g. metals) via diffusion gradients within the biofilm matrix [21,64], a benefit conferred to both single and multispecies cultures. With this, there is also the potential for community interactions (e.g. gene transfer) that play a role in an environmental consortia, but would confer significantly less benefit in single species biofilms [64]. Baker-Austin *et al*. [65] reviews explanations proposed for enhanced metal and antibiotic resistance in biofilms, including the biofilm being an ideal environment for lateral gene transfer (via plasmids, transposons, etc.). Although these mechanisms may contribute to the heightened metal tolerance of the OSPW community biofilms, it is important to note that species richness of the OSPW cultures was not tracked in our assay. Therefore, a few select metal tolerant species could have theoretically rescued the OSPW community (as defined in our assay), masking the effects the metals may have on the community as a whole. In other words, community tolerance is dependant on how the word “community” is defined. For example, a more stringent definition of the microbial community (i.e. required maintenance of the original species richness index) may result in comparatively lower metal tolerances than what is reported in this study, as death of highly susceptible species would quickly lower the overall community tolerance. Even so, metal concentrations reported in process water (S1 Table) were less than tolerances demonstrated by the OSPW consortia in our assay (S2 Table), most by multiple orders of magnitude. Thus, the decreased metal stress in the tailings pond environment would make any ‘rescuing effect’ from our assay inconsequential in an *in situ* biotechnological application of the endogenous community.

As discussed, the OSPW consortia had less significant correlations with physicochemical parameters, and those that did correlate significantly demonstrated weaker relationships overall (Tables 1–3). While these may be an effect of the aforementioned community interactions, we speculate that the addition of multiple bacterial species creates a more complex system in which correlations breakdown. This would be reflected in multiple metal-ion biochemical reactions available [16] due to the diversity of metabolisms in a multispecies community, that would otherwise be limited in a single species.

## Conclusions

Herein we assessed the metal susceptibility of a microbial community from an OSPW sample. The metal challenges were performed differently than previous studies [17,33–35] in that the inoculant was introduced directly into media containing the metal challenge. This approach allowed us to analyse planktonic survival and their pursuant capacity to attach and grow biofilms under metal stressors. This approach was chosen as we considered it more reflective of environmental conditions derived from a continuous release of metal pollutant challenge into the environment. Soft and borderline HSAB acids demonstrated the highest toxicity compared to hard acids, suggesting the significance of thiols and their relation to microbial sensitivities to xenobiotic metal species. We also evaluated the resistance data for correlations with physicochemical characteristics of each metal to provide insights into toxicology within the OSPW microbiome community. This approach highlights how a community versus single species of bacteria, while demonstrating comparable metal tolerance profiles, are affected differentially in their physiological sensitivity towards metals.

## Supporting Information

S1 Fig Linear regression analysis of minimum biofilm inhibitory concentrations (MBIC) of mixed species cultures derived from oil sands process water plotted against (A) metal-sulfide solubility product (pK_sp_), (B) standard reduction-oxidation potentials (ΔE_0_), (C) first ionization energy (*I*_*1*_), (D) electronegativity (X_m_), (E) Pearson’s softness index (σ_p_), and (F) first hydrolysis constants (|Log K_OH_|) of (i) all, (ii) Class I, and (iii) Class II metals.
(PDF)

S2 Fig Linear regression analysis of minimum inhibitory concentrations (MIC) of planktonic mixed species cultures derived from oil sands process water plotted against (A) metal-sulfide solubility product (pK_sp_), (B) standard reduction-oxidation potentials (ΔE_0_), (C) first ionization energy (*I*_*1*_), (D) electronegativity (X_m_), (E) Pearson’s softness index (σ_p_), and (F) first hydrolysis constants (|Log K_OH_|) of (i) all, (ii) Class I, and (iii) Class II metals.
(PDF)

S3 Fig Linear regression analysis of minimum biofilm inhibitory concentrations (MBIC) of *C*. *metallidurans* cultures plotted against (A) metal-sulfide solubility product (pK_sp_), (B) standard reduction-oxidation potentials (ΔE_0_), (C) first ionization energy (*I*_*1*_), (D) electronegativity (X_m_), (E) Pearson’s softness index (σ_p_), and (F) first hydrolysis constants (|Log K_OH_|) of (i) all, (ii) Class I, and (iii) Class II metals.
(PDF)

S4 Fig Linear regression analysis of minimum inhibitory concentrations (MIC) of planktonic *C*. *metallidurans* cultures plotted against (A) metal-sulfide solubility product (pK_sp_), (B) standard reduction-oxidation potentials (ΔE_0_), (C) first ionization energy (*I*_*1*_), (D) electronegativity (X_m_), (E) Pearson’s softness index (σ_p_), and (F) first hydrolysis constants (|Log K_OH_|) of (i) all, (ii) Class I, and (iii) Class II metals.
(PDF)

S1 Table Metals reported to be present in Syncrude Canada tailings ponds and process affected waters. Values are expressed as ranges of all data reported [8–10], blank cells indicate no data provided by the studies. Metal ranges were compiled from chemical characterization of Suncor’s Mildred Lake tailings ponds in 1980 (Apr–Oct, n>300) [10], constructed test ponds containing only mature fine tailings and/or tailings ponds water (SCL5, SCL9, and SCL10) [8], and samples of Suncor’s South Tailings Pond process affected water in 2008 and 2010 (PA 2008 and PA 2010, respectively) [9].
(PDF)

S2 Table Response of exposure of the OSPW consortia and *C*. *metallidurans* to metal ion exposure. Values are expressed (in mM) as means (± standard deviation) and ranges from the lowest and highest values obtained from two to nine trials. Data with no range indicated had the same value across all trials, or inhibitory concentrations were not reached (i.e. Mg > 250). MIC: Minimum inhibitory concentration; MBIC: minimum biofilm inhibitory concentration; MIC* values obtained from [31].
(PDF)

S3 Table Metal ion-specific physicochemical parameters. Class indicates grouping according to orbital structure, as described in [52]. Molecular ions that could not be classified according to this were left blank. Electronegativity (X_m_), standard reduction-oxidation potentials (ΔE_0_), and first ionization energy (*I*_*1*_) were obtained from the CRC Handbook of Chemistry and Physics [66]. The Pearson’s softness index (σ_p_) is defined as the coordinate bond energy of the metal fluoride minus the coordinate bond energy of the metal iodide, divided by the coordinate bond energy of the metal fluoride; these values were obtained from [67]. Hard-soft acid base designations (HA–hard acid; SA–soft acid; BA–borderline acid) were taken from [57]. Values for the metal-sulfide solubility product (pK_sp_) were obtained from [59]. The first hydrolysis constants (|Log K_OH_|) were obtained from [68], and defined as the formation constant for the first hydrolysis product produced by the metals reaction with water (K_OH_ for M^n+^ + H_2_O → MOH^n-1^ +H^+^).
(PDF)

S4 Table Metal susceptibility data set for OSPW community cultures, reported (in mM) as minimum biofilm inhibitory concentration (MBIC) and planktonic minimum inhibitory concentration (MIC). Values in blue indicate metal tolerances above detectable limits of the assay.
(PDF)

S5 Table Metal susceptibility data set for *C. metallidurans* cultures, reported (in mM) as minimum biofilm inhibitory concentration (MBIC) and planktonic minimum inhibitory concentration (MIC). Values in blue indicate metal tolerances above detectable limits of the assay.
(PDF)
