# Supplementary figures and images for: Evaluating the Metal Tolerance Capacity of Microbial Communities Isolated from Alberta Oil Sands Process Water
Source: PLoS One. 2016 Feb 5;11(2):e0148682. doi: 10.1371/journal.pone.0148682 (PMC4743850; doi:10.1371/journal.pone.0148682)

## All Metals

## Class I

## Class II

A

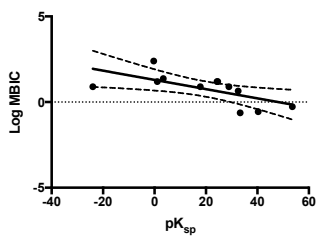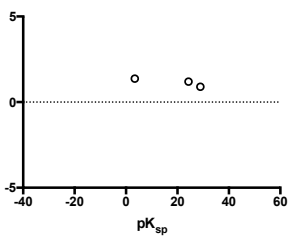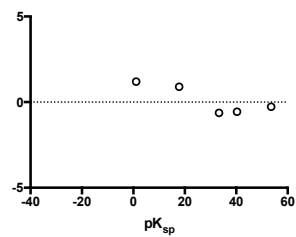

B

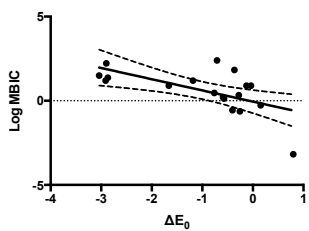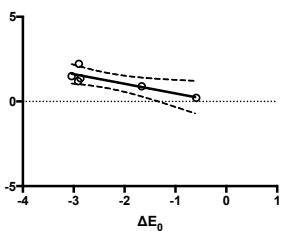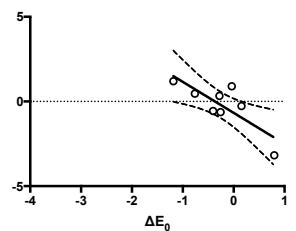

C

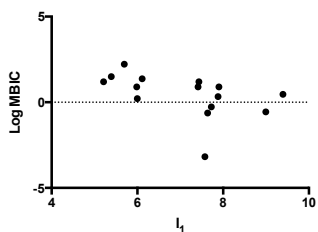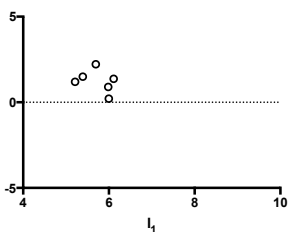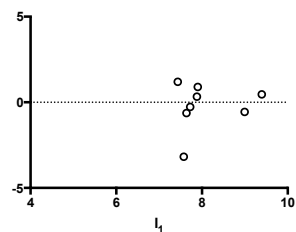

D

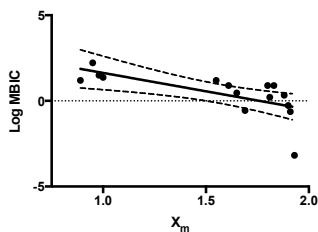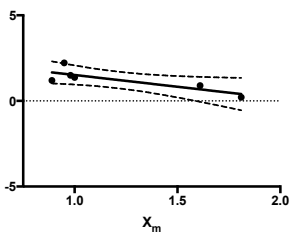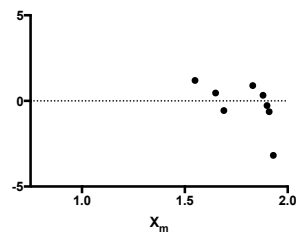

E

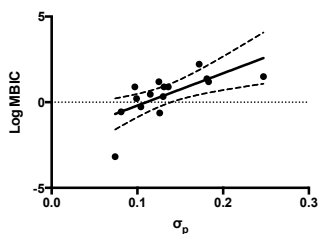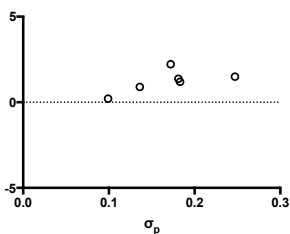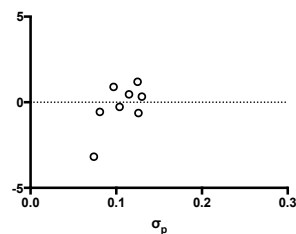

F

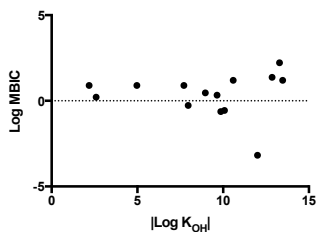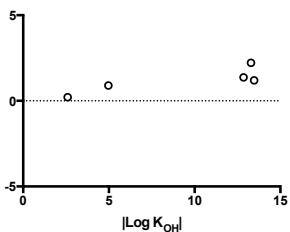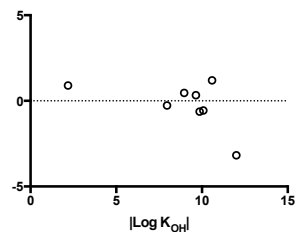

i

ii

iii

Supplement: S1 Fig — Linear regression analysis of minimum biofilm inhibitory concentrations (MBIC) of mixed species cultures derived from oil sands process water plotted against (A) metal-sulfide solubility product (pKsp), (B) standard reduction-oxidation potentials (ΔE0), (C) first ionization energy (I1), (D) electronegativity (Xm), (E) Pearson’s softness index (σp), and (F) first hydrolysis constants (|Log KOH|) of (i) all, (ii) Class I, and (iii) Class II metals. (PDF) [file pone.0148682.s001.pdf]

## All Metals

## Class I

## Class II

A

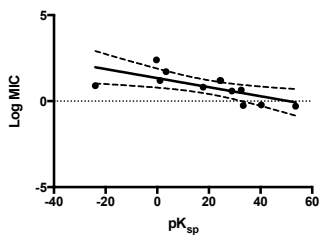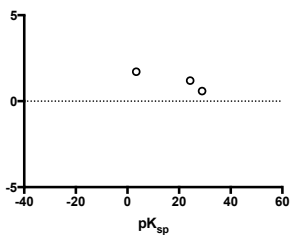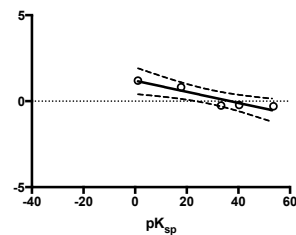

B

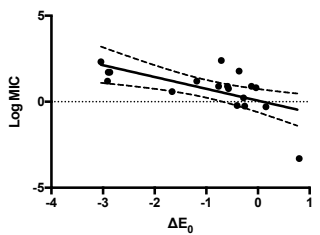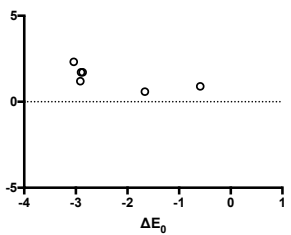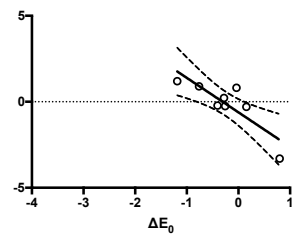

C

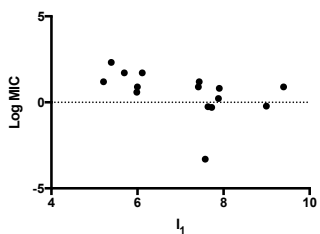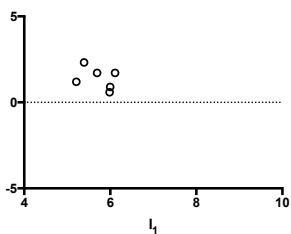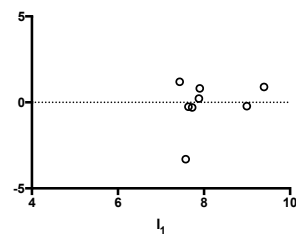

D

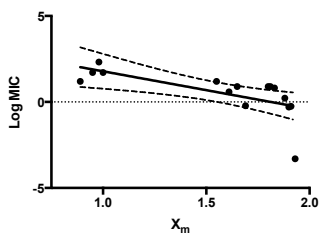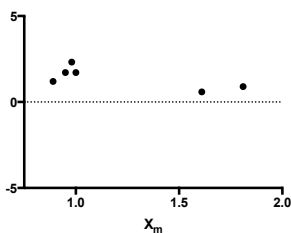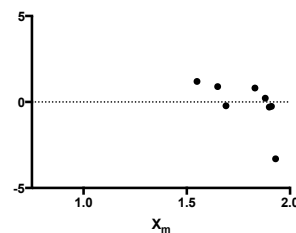

E

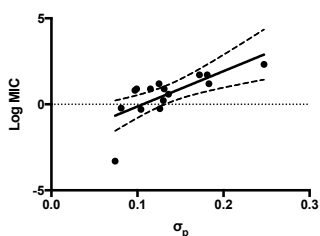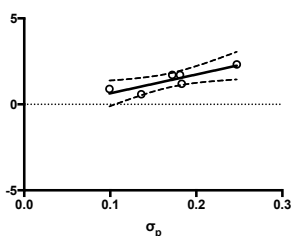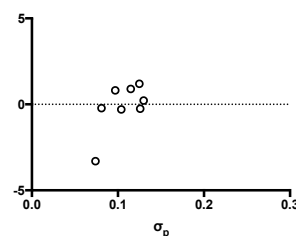

F

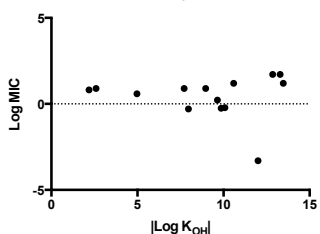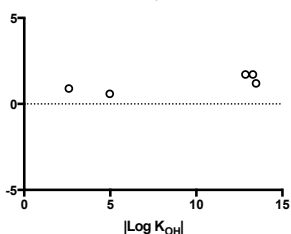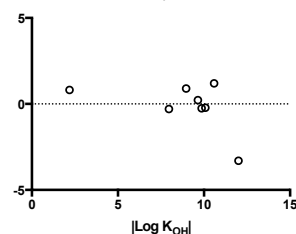

i

ii

iii

Supplement: S2 Fig — Linear regression analysis of minimum inhibitory concentrations (MIC) of planktonic mixed species cultures derived from oil sands process water plotted against (A) metal-sulfide solubility product (pKsp), (B) standard reduction-oxidation potentials (ΔE0), (C) first ionization energy (I1), (D) electronegativity (Xm), (E) Pearson’s softness index (σp), and (F) first hydrolysis constants (|Log KOH|) of (i) all, (ii) Class I, and (iii) Class II metals. (PDF) [file pone.0148682.s002.pdf]

## All Metals

## Class I

## Class II

A

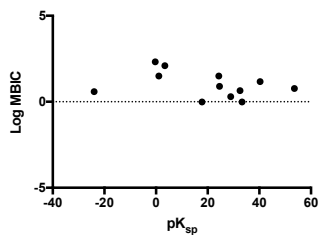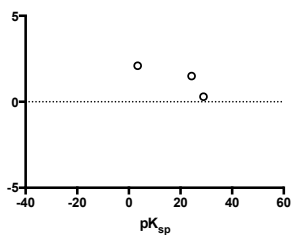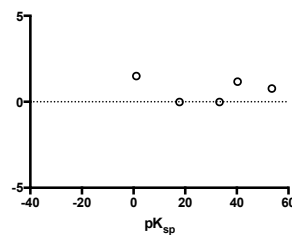

B

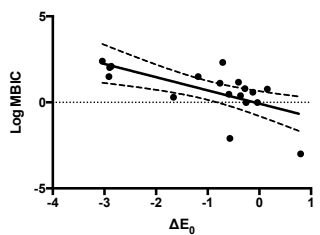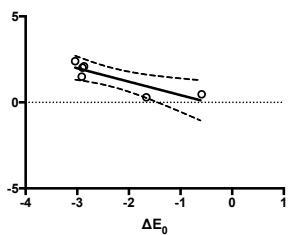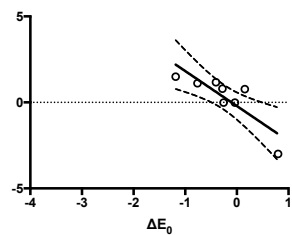

C

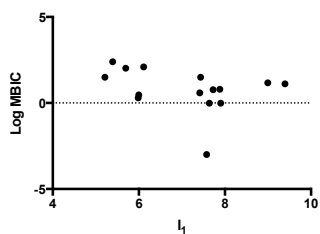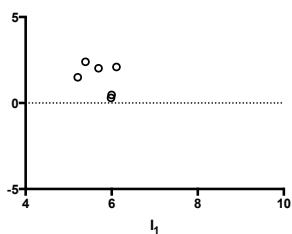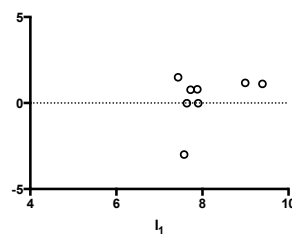

D

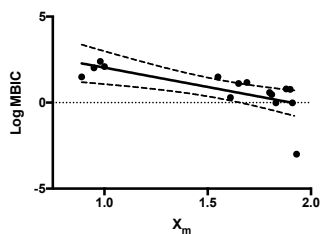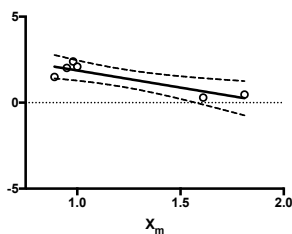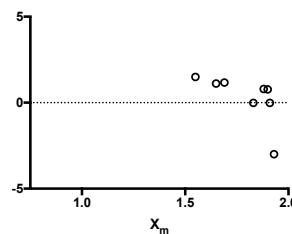

E

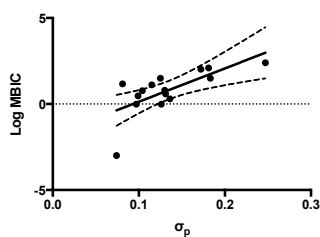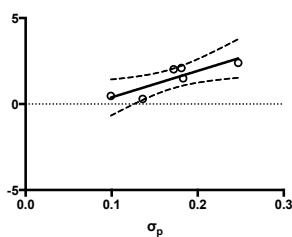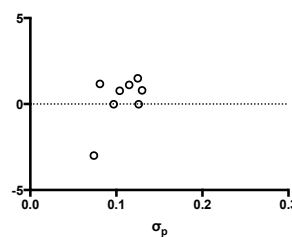

F

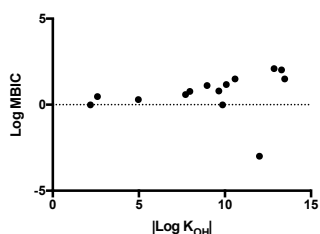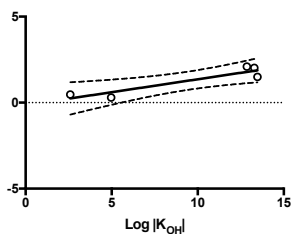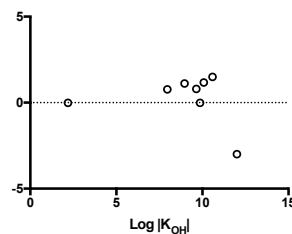

i

ii

iii

Supplement: S3 Fig — Linear regression analysis of minimum biofilm inhibitory concentrations (MBIC) of C. metallidurans cultures plotted against (A) metal-sulfide solubility product (pKsp), (B) standard reduction-oxidation potentials (ΔE0), (C) first ionization energy (I1), (D) electronegativity (Xm), (E) Pearson’s softness index (σp), and (F) first hydrolysis constants (|Log KOH|) of (i) all, (ii) Class I, and (iii) Class II metals. (PDF) [file pone.0148682.s003.pdf]

# All Metals

# Class I

# Class II

**A**

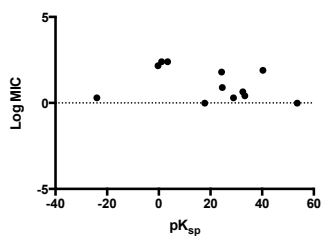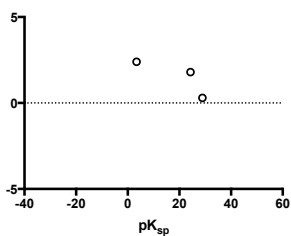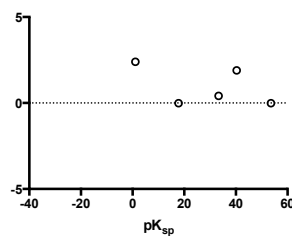

**B**

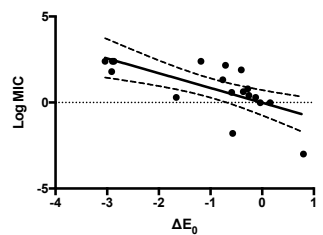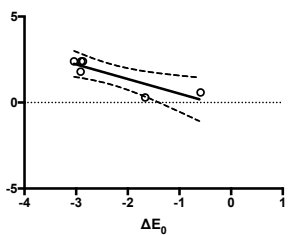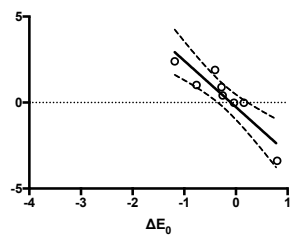

**C**

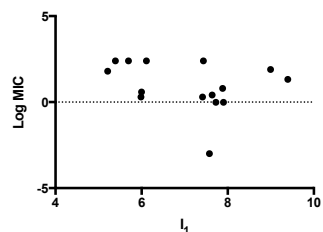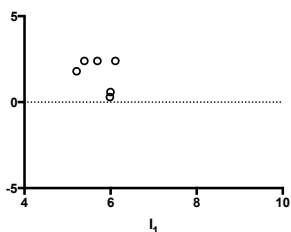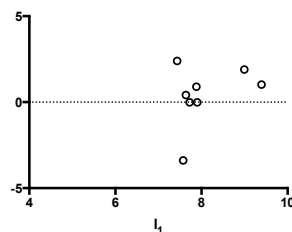

**D**

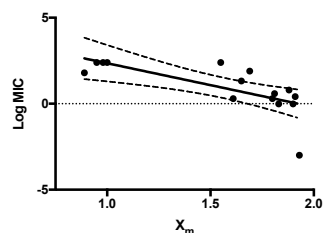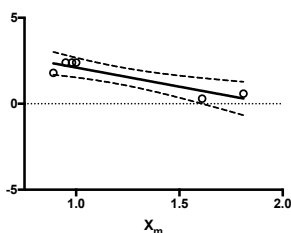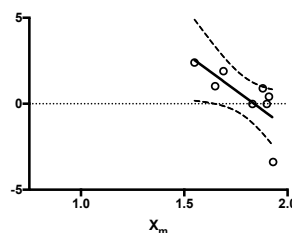

**E**

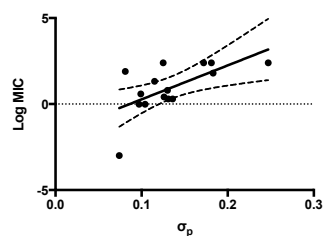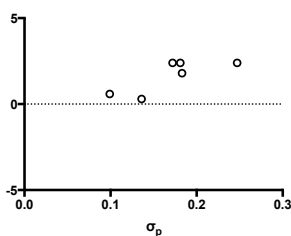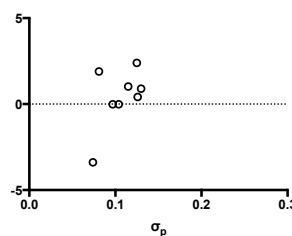

**F**

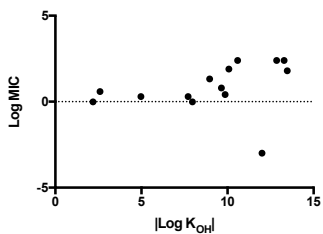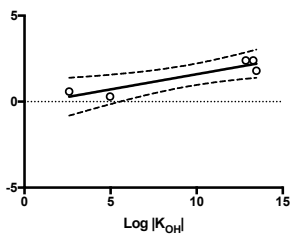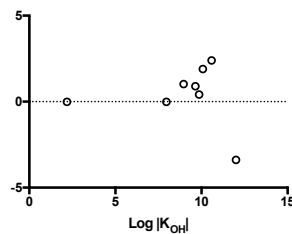

**i**

**ii**

**iii**

Supplement: S4 Fig — Linear regression analysis of minimum inhibitory concentrations (MIC) of planktonic C. metallidurans cultures plotted against (A) metal-sulfide solubility product (pKsp), (B) standard reduction-oxidation potentials (ΔE0), (C) first ionization energy (I1), (D) electronegativity (Xm), (E) Pearson’s softness index (σp), and (F) first hydrolysis constants (|Log KOH|) of (i) all, (ii) Class I, and (iii) Class II metals. (PDF) [file pone.0148682.s004.pdf]
