# Supplementary material for: Evaluating the Metal Tolerance Capacity of Microbial Communities Isolated from Alberta Oil Sands Process Water
Source: PLoS One. 2016 Feb 5;11(2):e0148682. doi: 10.1371/journal.pone.0148682 (PMC4743850; doi:10.1371/journal.pone.0148682)
Supplement: S1 Table — Values are expressed as ranges of all data reported [8–10], blank cells indicate no data provided by the studies. Metal ranges were compiled from chemical characterization of Suncor’s Mildred Lake tailings ponds in 1980 (Apr–Oct, n>300) [10], constructed test ponds containing only mature fine tailings and/or tailings ponds water (SCL5, SCL9, and SCL10) [8], and samples of Suncor’s South Tailings Pond process affected water in 2008 and 2010 (PA 2008 and PA 2010, respectively) [9]. (PDF) [file pone.0148682.s005.pdf]

Metal	Range (mM)
Li	0.012 - 0.030
Mg	0.0749 - 1.127
Ca	0.3069 - 1.422
Sr	0.000002 - 0.0015
Ba	0.00004 - 0.00047
Al	0.002 - 2.532
Ga	
Fe	0.001 - 0.8333
Ag	
Cd	0.00002 - 0.0040
Mn	0.000002 - 0.0085
Co	0.00005 - 0.00006
Ni	0.00009 - 0.0001
Cu	0.00002 - 0.00006
Zn	0 - 0.00016
Pb	0.000003 - 0.0002
V	< 0.00004
Mo	0.00005 - 0.0157
W	
As	0.00004 - 0.00008
Te	
Se	
