# Supplementary material for: Evaluating the Metal Tolerance Capacity of Microbial Communities Isolated from Alberta Oil Sands Process Water
Source: PLoS One. 2016 Feb 5;11(2):e0148682. doi: 10.1371/journal.pone.0148682 (PMC4743850; doi:10.1371/journal.pone.0148682)
Supplement: S2 Table — Values are expressed (in mM) as means (± standard deviation) and ranges from the lowest and highest values obtained from two to nine trials. Data with no range indicated had the same value across all trials, or inhibitory concentrations were not reached (i.e. Mg > 250). MIC: Minimum inhibitory concentration; MBIC: minimum biofilm inhibitory concentration; MIC* values obtained from [31]. (PDF) [file pone.0148682.s006.pdf]

Metal	OSPW Community		*C. metallidurans*		
	MICY	MBICY	MICY	MBICY	MIC*
Li	208 ± 72 (125 - 250)	31.2	>250	250	
Mg	>250	>250	≥250	208 ± 72.2 (125 - 250)	
Ca	52.1 ± 18.1 (31.2 - 62.5)	23.4 ± 13.5 (7.8 - 31.2)	>250	125	
Sr	52.1 ± 18.1 (31.2 - 62.5)	167 ± 72 (125 - 250)	250	104 ± 36 (62.5 - 250)	
Ba	15.6	15.6	62.5	31.2	
Al	3.9	7.8	1.96	1.96	
Ga	7.8	1.63 ± 0.57 (0.98 - 1.96)	3.9	2.93 ± 1.12 (1.96 - 3.9)	
Fe	6.5 ± 2.3 (3.9 - 7.8)	7.8	0.98	0.98	
Ag	0.0005	0.0007 ± 0.0002 (0.0005 - 0.001)	0.001	0.001	0.0003
Cd	0.60 ± 0.57 (0.08 - 1.6)	0.27 ± 0.24 (0.05 - 0.62)	78.1 ± 38.3 (31.2 - 125)	15 ± 13.4 (3.9 - 31.2)	4
Mn	15.6	15.6	250	31.2	25
Co	1.65 ± 1.70 (0.1 - 3.2)	2.12 ± 2.53 (0.02 - 6.2)	6.2	6.2	25
Ni	0.56 ± 0.28 (0.2 - 0.98)	0.23 ± 0.15 (0.1 - 0.4)	2.6 ± 1.1 (1.96 - 3.9)	0.98	13
Cu	0.51 ± 0.15 (0.4 - 0.8)	0.53 ± 0.23 (0.4 - 0.8)	0.98	5.85 ± 8.44 (0.98 - 15.6)	3
Zn	7.8	2.9 ± 1.7 (0.98 - 3.9)	20.8 ± 9.0 (15.6 - 31.2)	13 ± 4.5 (7.8 - 15.6)	12
Pb	7.8	7.8	1.96	3.9	1
V	>4.4	>4.4	>4.4	≥4.4	
Mo	15.6	15.6	7.8	7.8	
W	23.4 ± 11.0 (15.6 - 31.2)	78.1 ± 66.3 (31.2 - 125)	125	208 ± 72 (125 - 250)	
As	>250	>250	145 ± 96 (62.5 - 250)	208 ± 72 (125 - 250)	
Te	5.83 ± 10.7 (0.4 - 25)	1.35 ± 0.56 (0.8 - 1.96)	0.016	0.008	
Se	60 ± 40.8 (25 - 125)	67.5 ± 56.3 (12.5 - 125)	4.26 ± 3.5 (0.1 - 7.8)	2.43 ± 1.15 (1.6 - 3.9)	
