# Supplementary material for: Evaluating the Metal Tolerance Capacity of Microbial Communities Isolated from Alberta Oil Sands Process Water
Source: PLoS One. 2016 Feb 5;11(2):e0148682. doi: 10.1371/journal.pone.0148682 (PMC4743850; doi:10.1371/journal.pone.0148682)
Supplement: S3 Table — Class indicates grouping according to orbital structure, as described in [52]. Molecular ions that could not be classified according to this were left blank. Electronegativity (Xm), standard reduction-oxidation potentials (ΔE0), and first ionization energy (I1) were obtained from the CRC Handbook of Chemistry and Physics [66]. The Pearson’s softness index (σp) is defined as the coordinate bond energy of the metal fluoride minus the coordinate bond energy of the metal iodide, divided by the coordinate bond energy of the metal fluoride; these values were obtained from [67]. Hard-soft acid base designations (HA–hard acid; SA–soft acid; BA–borderline acid) were taken from [57]. Values for the metal-sulfide solubility product (pKsp) were obtained from [59]. The first hydrolysis constants (|Log KOH|) were obtained from [68], and defined as the formation constant for the first hydrolysis product produced by the metals reaction with water (KOH for Mn+ + H2O → MOHn-1 +H+). (PDF) [file pone.0148682.s007.pdf]

Salt	Metal	Oxidation No.	Class (a)	HSAB	pK_{sp}	ΔE0	I_1	X_m	σ_p	|log KOH|
LiCl	Li	[I]	I	HA		-3.0401	5.391719	0.98	0.247	
$MgSO_4$	Mg	[II]	I	HA	-0.3	-2.372	7.646235	1.31	0.167	
CaCl2	Ca	[II]	I	HA		-2.868	6.11316	1.00	0.181	12.85
$SrCl_2$	Sr	[II]	I	HA	3.4	-2.899	5.69485	0.95	0.172	13.29
BaO	Ba	[II]	I	HA	1.1	-2.912	5.211664	0.89	0.183	13.47
$Al_2(SO_4)_3 \cdot xH_2O$	Al	[III]	I	HA	-24	-1.662	5.985768	1.61	0.136	4.97
Ga(NO3)3	Ga	[III]	I	HA		-0.589	5.999301	1.81	0.099	2.6
$Fe_2(SO_4)_3$	Fe	[III]	II	HA		-0.037	7.9024	1.83	0.097	2.19
AgNO3	Ag	[I]	II	SA	53.6	0.7996	7.57623	1.93	0.074	12
$3CdSO_4 \cdot 8H_2O$	Cd	[II]	II	SA	33.3	-0.403	8.99382	1.69	0.081	10.08
MnSO4·H2O	Mn	[II]	II	BA	17.8	-1.185	7.43402	1.55	0.125	10.59
$CoCl_2$	Co	[II]	II	BA	24.6	-0.28	7.88101	1.88	0.130	9.65
NiCl2·6H2O	Ni	[II]	II	BA	24.3	-0.257	7.6398	1.91	0.126	9.86
$CuSO_4 \cdot 5H_2O$	Cu	[II]	II	BA	40.3	0.153	7.72638	1.90	0.104	7.96
ZnCl2	Zn	[II]	II	BA	28.9	-0.7618	9.394199	1.65	0.115	8.96
$Pb(NO_3)_2$	Pb	[II]	III	BA	32.5	-0.1262	7.41663	1.80	0.131	7.71
V2O5	V	[V]								
$(NH_4)_6Mo_7O_{24} \cdot 4H_2O$	Mo	[VI]								
Na2WO4·2H2O	W	[VI]								
$Na_2HAsO_4 \cdot 7H_2O$	As	[V]				-0.71				
K2TeO3·xH2O	Te	[IV]				-0.57				
Na_2SeO_3	Se	[IV]				-0.366				
