# Supplementary material for: Evaluating the Metal Tolerance Capacity of Microbial Communities Isolated from Alberta Oil Sands Process Water
Source: PLoS One. 2016 Feb 5;11(2):e0148682. doi: 10.1371/journal.pone.0148682 (PMC4743850; doi:10.1371/journal.pone.0148682)
Supplement: S4 Table — Values in blue indicate metal tolerances above detectable limits of the assay. (PDF) [file pone.0148682.s008.pdf]

Metal susceptability data set for OSPW community cultures, reported (in mM) as minimum biofilm inhibitory concentration (MBIC) and planktonic minimum inhibitory concentration (MIC). Values in blue indicate metal tolerances above detectabe limits of the assay.

Metal	MBIC						MIC					
Li	31.2	31.2	31.2				125	250	250			
Mg	>250	>250	>250				>250	>250	>250			
Ca	31.2	7.8	31.2				31.2	62.5	62.5			
Sr	125	250	125				62.5	31.2	62.5			
Ba	15.6	15.6	15.6				15.6	15.6	15.6			
Al	7.8	7.8	7.8				3.9	3.9	3.9			
Ga	1.96	1.96	0.98				7.8	7.8	7.8			
Fe	7.8	7.8	7.8				7.8	7.8	3.9			
Ag	0.0005	0.001	0.0005				0.0005	0.0005	0.0005			
Cd	0.4	0.05	0.4	0.08	0.08	0.62	1.6	0.4	0.8	0.08	0.08	0.62
Mn	15.6	15.6	15.6				15.6	15.6	15.6			
Co	6.2	3.2	3.2	0.02	0.02	0.1	3.2	3.2	3.2	0.1	0.1	0.1
Ni	0.4	0.2	0.1				0.2	0.8	0.4	0.48	0.48	0.98
Cu	0.8	0.4	0.4				0.8	0.4	0.4	0.48	0.48	0.48
Zn	3.9	3.9	0.98				7.8	7.8	7.8			
Pb	7.8	7.8	7.8				7.8	7.8	7.8			
V	>4.4	>4.4	>4.4				>4.4	>4.4	>4.4			
Mo	15.6	15.6					15.6	15.6				
W	31.2	125					31.2	15.6				
As	>250	>250	>250				>250	>250	>250			
Te	1.96	0.98	1.96	0.8	1.6	0.8	0.4	0.8	25	0.98	1.96	
Se	125	125	62.5	12.5	12.5		25	25	62.5	125	62.5	
