# Supplementary material for: Evaluating the Metal Tolerance Capacity of Microbial Communities Isolated from Alberta Oil Sands Process Water
Source: PLoS One. 2016 Feb 5;11(2):e0148682. doi: 10.1371/journal.pone.0148682 (PMC4743850; doi:10.1371/journal.pone.0148682)
Supplement: S5 Table — Values in blue indicate metal tolerances above detectable limits of the assay. (PDF) [file pone.0148682.s009.pdf]

Metal susceptability data set for *C. metallidurans* cultures, reported (in mM) as minimum biofilm inhibitory concentration (MBIC) and planktonic minimum inhibitory concentration (MIC). Values in blue indicate metal tolerances above detectabe limits of the assay.

Metal	MBIC						MIC					
Li	250	250	250				>250	>250	>250			
Mg	250	125	250				>250	>250	250			
Ca	125	125	125				>250	>250	>250			
Sr	62.5	125	125				250	250	250			
Ba	31.2	31.2	31.2				62.5	62.5	62.5			
Al	1.96	1.96	1.96				1.96	1.96	1.96			
Ga	1.96	1.96	3.9	3.9			3.9	3.9	3.9	3.9	3.9	3.9
Fe	0.98	0.98	0.98				0.98	0.98	0.98			
Ag	0.001	0.001	0.001				0.001	0.001	0.001			
Cd	3.9	3.9	3.9	31.2	31.2	15.6	62.5	62.5	62.5	31.2	125	125
Mn	31.2	31.2	31.2				250	250	250			
Co	6.2	6.2					6.2	6.2	6.2			
Ni	0.98	0.98	0.98				1.96	3.9	1.96			
Cu	0.98	0.98	15.6				0.98	0.98	0.98			
Zn	7.8	15.6	15.6				15.6	15.6	31.2			
Pb	3.9	3.9	3.9				1.96	1.96	1.96			
V	4.4	4.4	>4.4				>4.4	>4.4	>4.4			
Mo	7.8	7.8	7.8				7.8	7.8	7.8			
W	125	250	250				125	125	125			
As	125	250	250				250	125	62.5			
Te	0.008	0.008	0.008				0.016	0.016	0.016			
Se	3.9	3.9	1.96	1.6	1.6	1.6	0.48	7.8	7.8	3.2	0.1	6.2
